# Chemical and antibacterial data of synthesized thioureido derivatives

**DOI:** 10.1016/j.dib.2019.104651

**Published:** 2019-10-16

**Authors:** M.A. Kadir, R. Ramli, M.S.M. Yusof, N. Ismail, N. Ngah, N.S.H. Haris

**Affiliations:** a Faculty of Science and Marine Environment, Universiti Malaysia Terengganu, 21030, Kuala Nerus, Terengganu, Malaysia; b Institute of Marine Biotechnology, Universiti Malaysia Terengganu, 21030, Kuala Nerus, Terengganu, Malaysia; c Department of Chemistry, Kulliyah of Science, International Islamic University Malaysia, Kuantan Campus, 25200, Kuantan, Pahang, Malaysia

**Keywords:** Spectroscopic, Thioureido, Amino acid, Antibacterial

## Abstract

This paper provided comprehensive data on spectroscopic and antibacterial activities of thioureido compounds which are relevant with research article entitled “Synthesis, Spectroscopic Studies and Antibacterial Activity of New Lauroyl Thiourea Amino Acid Derivatives” [1]. Based on the reported study, four new thioureido derivatives, namely 3-(3-dodecanoyl-thioureido)propionic acid (R1), 2-(3-dodecanoyl-thioureido)-3-methyl butyric acid (R2), (3-dodecanoyl-thioureido)acetic acid (R3) and 2-(3-dodecanoyl-thioureido)-3-phenyl propionic acid (R4) were characterized by elemental analysis, Fourier Transform Infrared (FTIR), ^1^H Nuclear Magnetic Resonance (^1^H NMR) and ^13^C Nuclear Magnetic Resonance (^13^C NMR), and Ultraviolet Visible spectroscopy (UV-Vis). The preliminary results from antibacterial assay which were tested against Gram-positive bacteria such as *Bacillus subtilis*, *Staphylococcus epidermidis, Staphylococcus aureus* and Gram-negative bacteria such as *Escherichia coli, Salmonella typhimurium* are also described.

**Table:** Specifications Table

Subject area	*Chemistry*
More specific subject area	*Synthetic chemistry, spectroscopy*
Type of data	*FTIR spectra, NMR spectra, UV spectra, graph, table,*
How data was acquired	*CHNS Analyzer Flashea 1112 series, FTIR Perkin Elmer Spectrum 100 and the spectra was recorded in range of 4000-*400 cm^−1^*utilizing potassium bromide (KBr) pellet, Spectrophotometer Shimadzu UV-1800, Bruker Avance II 400 spectrometer was used to record the*^*1*^*H and*^*13*^*C Nuclear Magnetic Resonance*
Data format	*JPEG, Tiff (Raw)*
Experimental factors	*Streptomycin (Abtek Biologicals Ltd) was used as the positive control while methanol served as negative control.*
Experimental features	*All chemicals used were commercially available and used as received without purification.*
Data source location	*Universiti Malaysia Terengganu*
Data accessibility	*Data is included with this article*
Related research article	*M.A. Kadir, R. Ramli, M.S.M. Yusof, N. Ismail, N. Ngah, Synthesis, Spectroscopic Studies and Antibacterial Activity of New Lauroyl Thiourea Amino Acid Derivatives, Asian Journal of Chemistry 28 (2016) 596–600.* [1]

**Table:** 

**Value of the Data** • The proposed synthetic methods is facile and highly recommended for the synthesis of new thioureido derivatives. • Thioureido derivatives with long alkyl chains have good potential for use in pharmacological area mainly as antibacterial and anticancer agents. • The spectroscopic data information can be manipulated for advanced molecular studies of biological active molecules.

## 1 Data

**Fig. 1:**
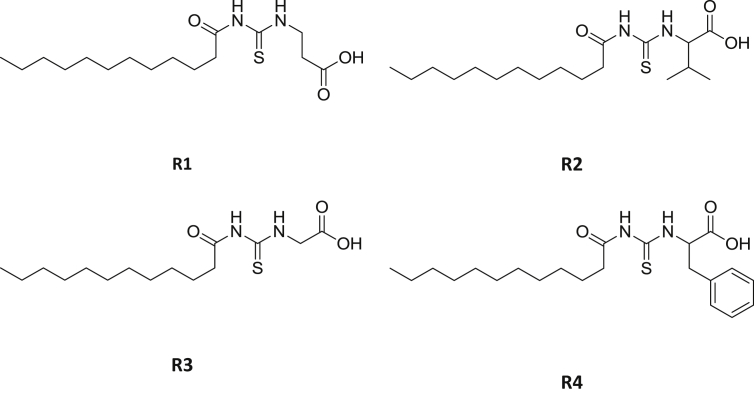
The structure of R1, R2, R3 and R4.

**Fig. 2:**
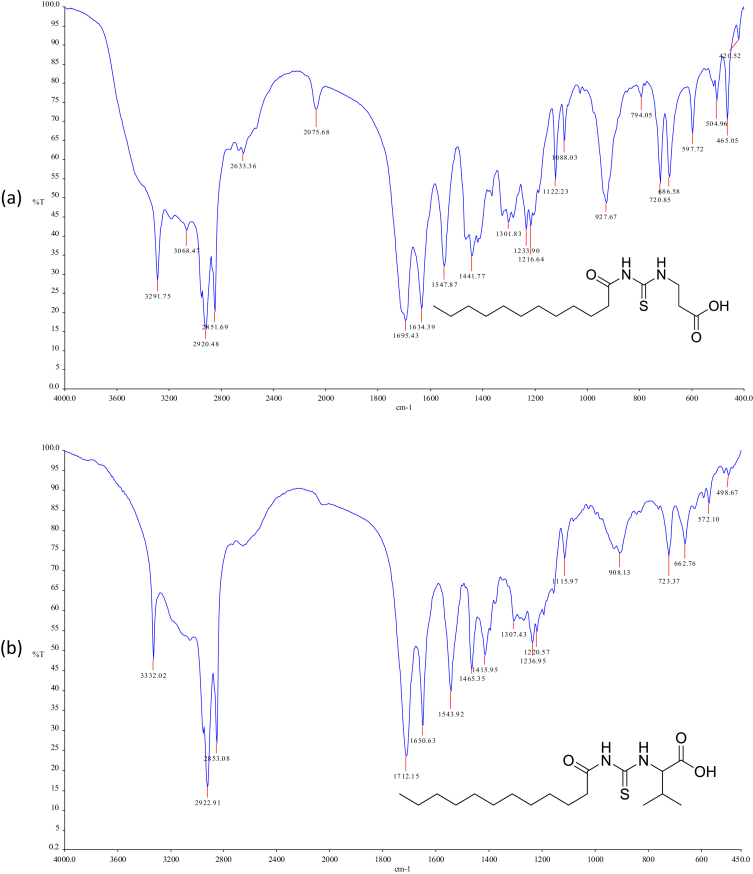

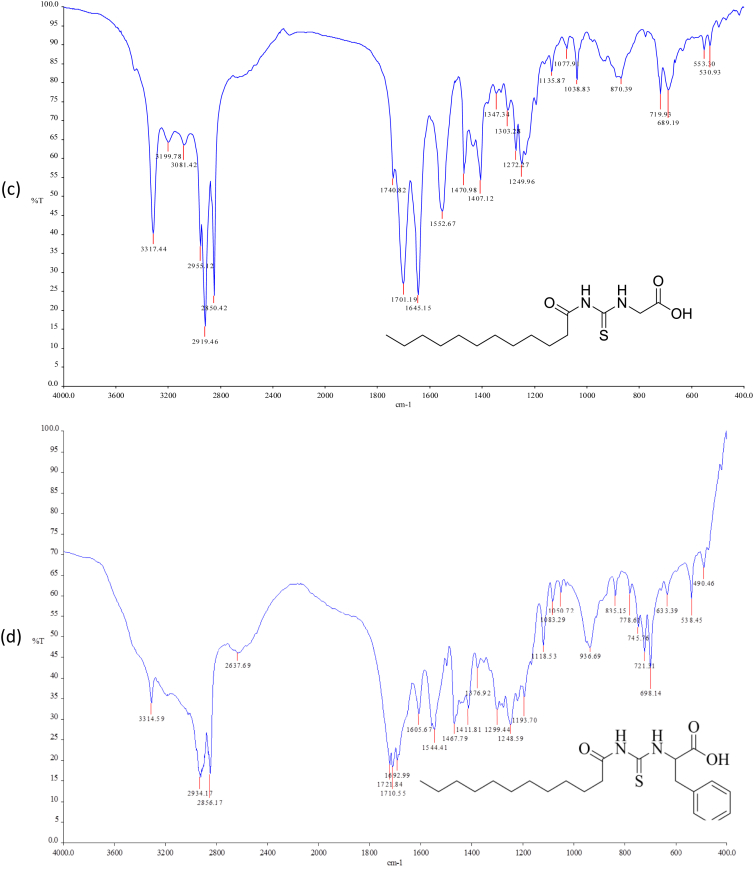
FTIR spectra of (a) R1 (b) R2 (c) R3 (d) R4.

**Fig. 3:**
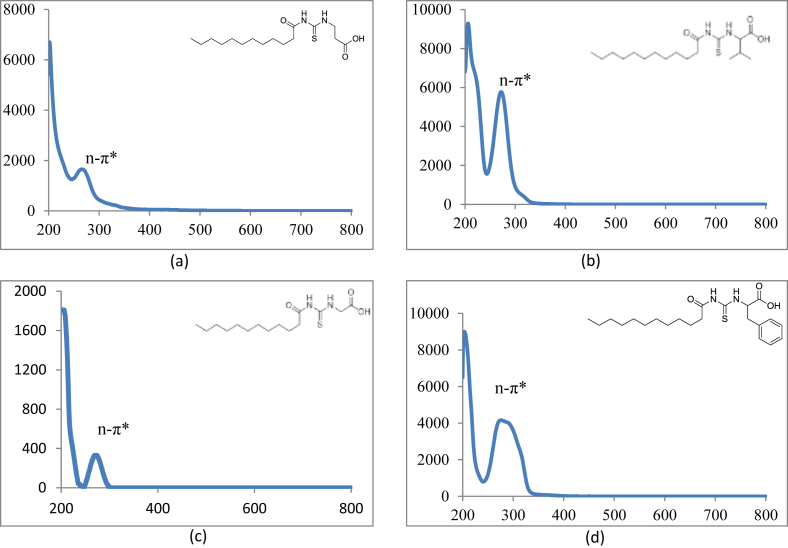
UV spectra of (a) R1 (b) R2,(c) R3,(d) R4 in methanol solution.

**Fig. 4:**
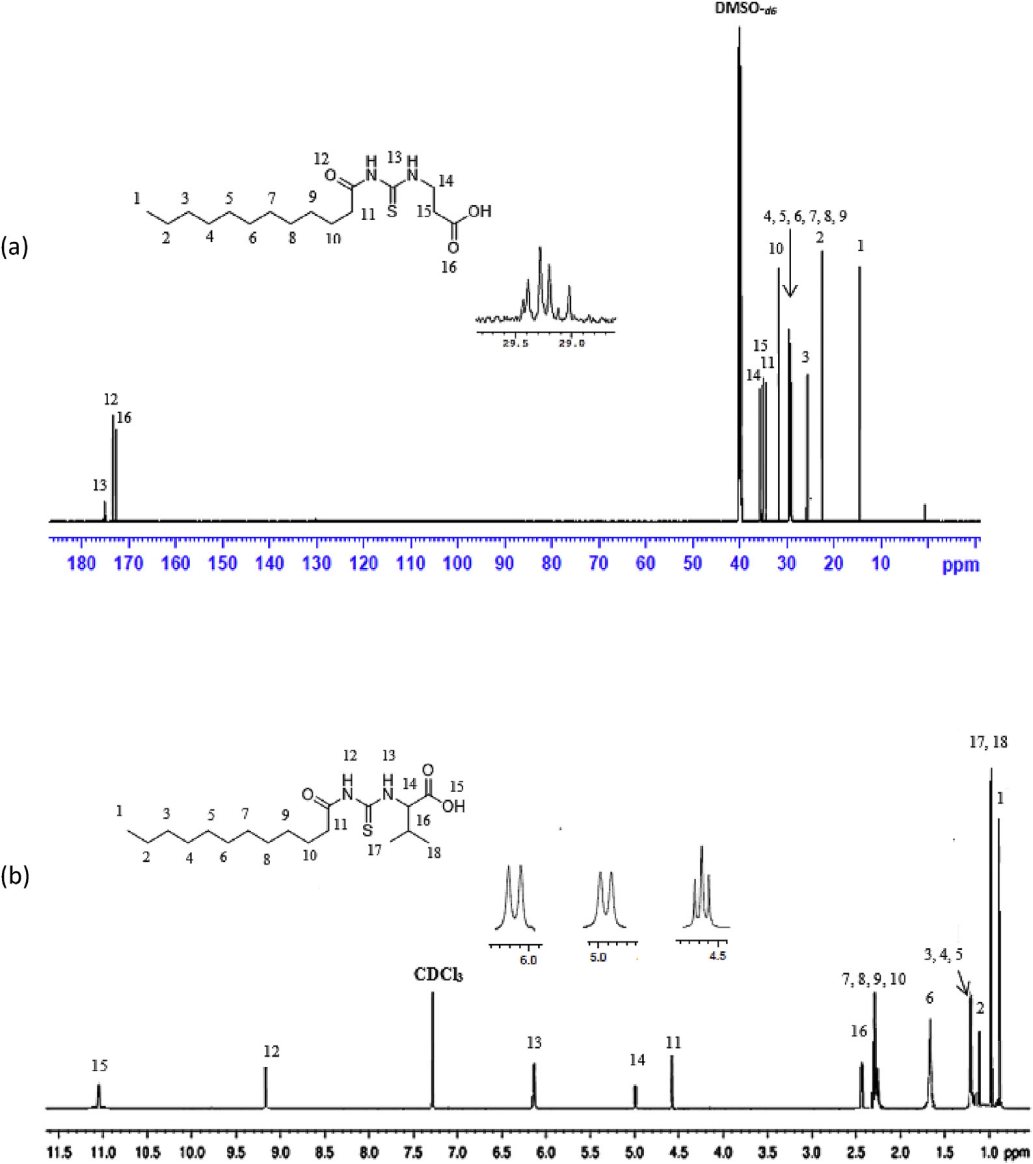

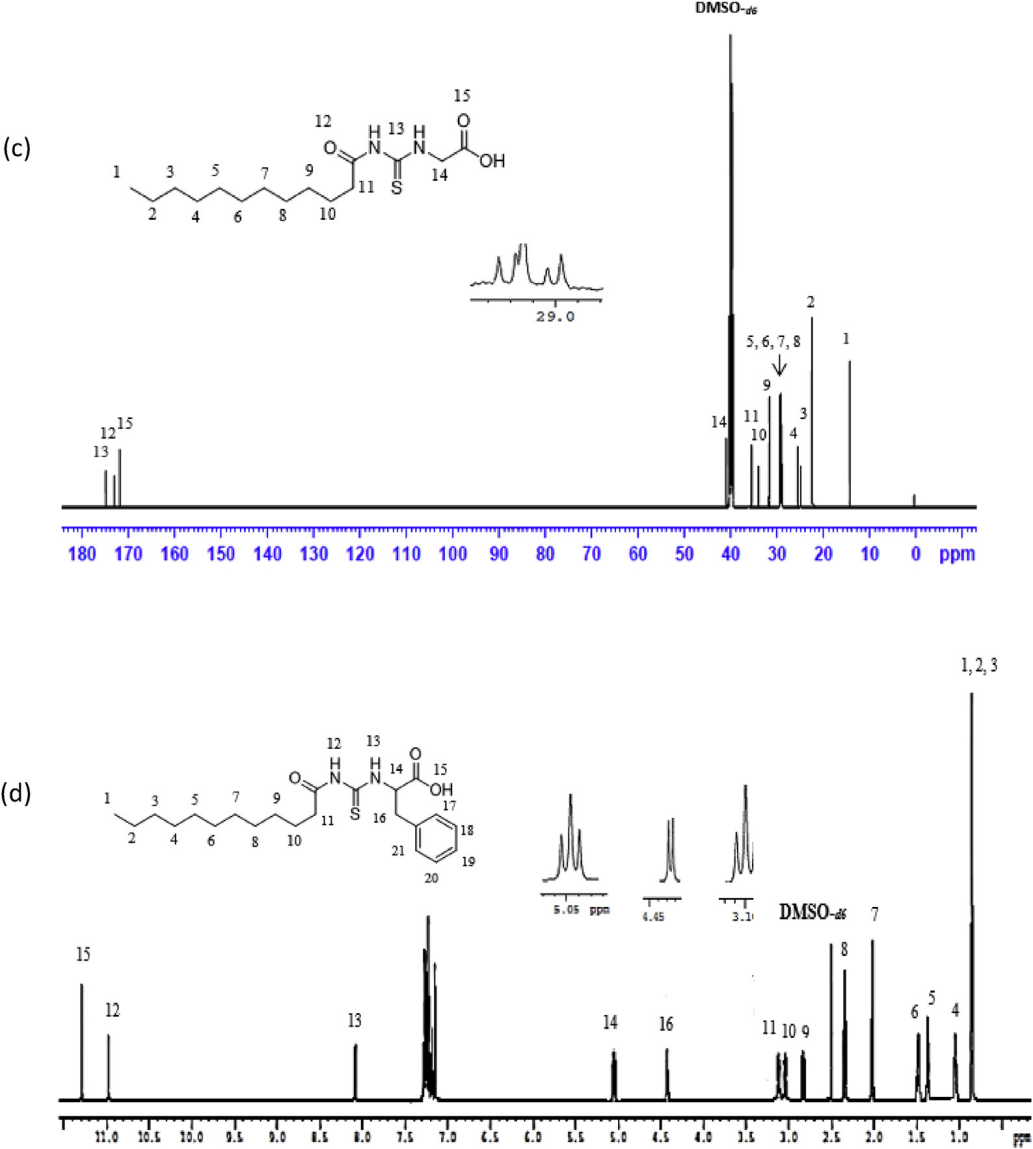
^1^H NMR spectra for (a) R1 (b) R2 (c) R3 and (d) R4.

**Fig. 5:**
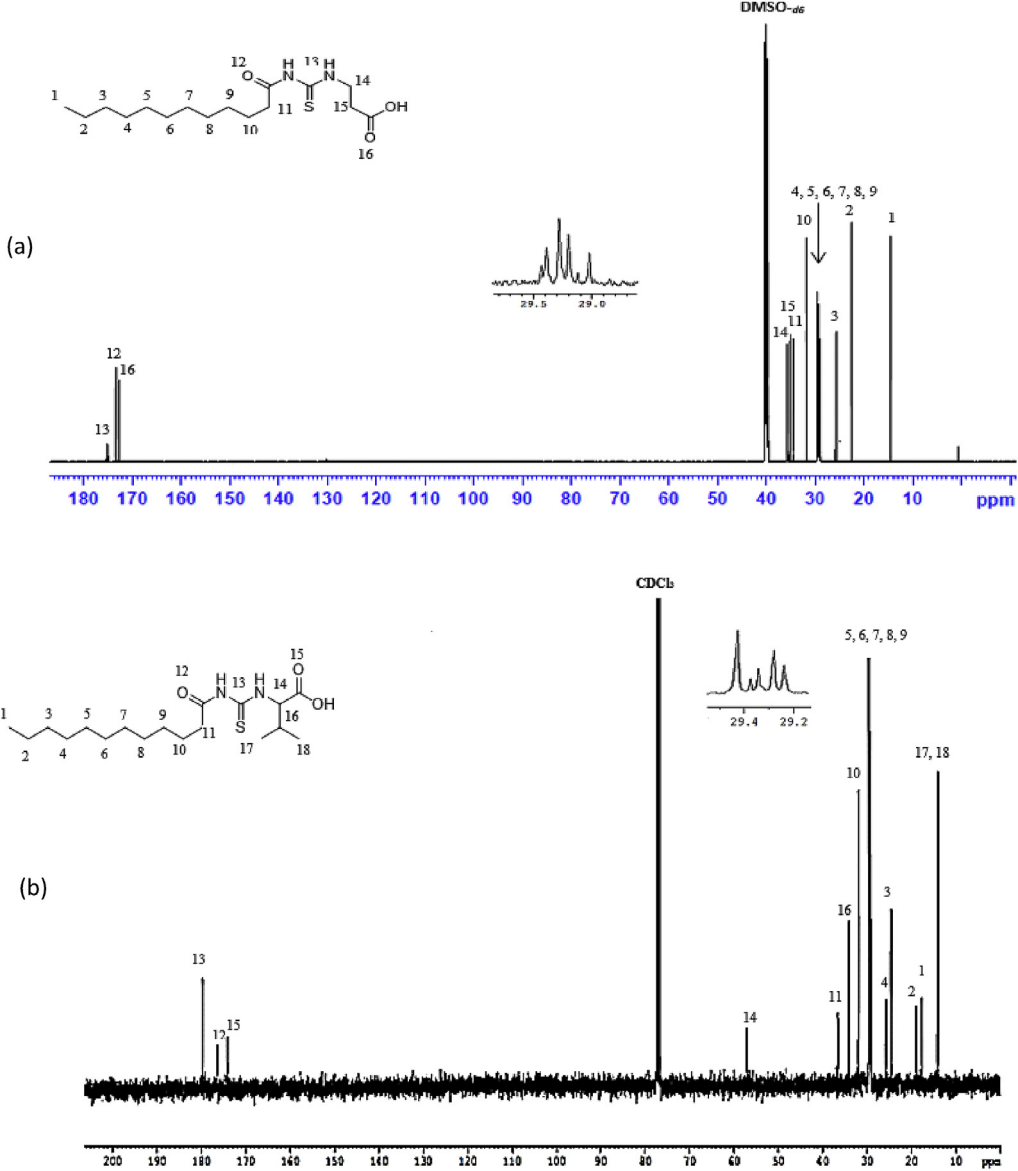

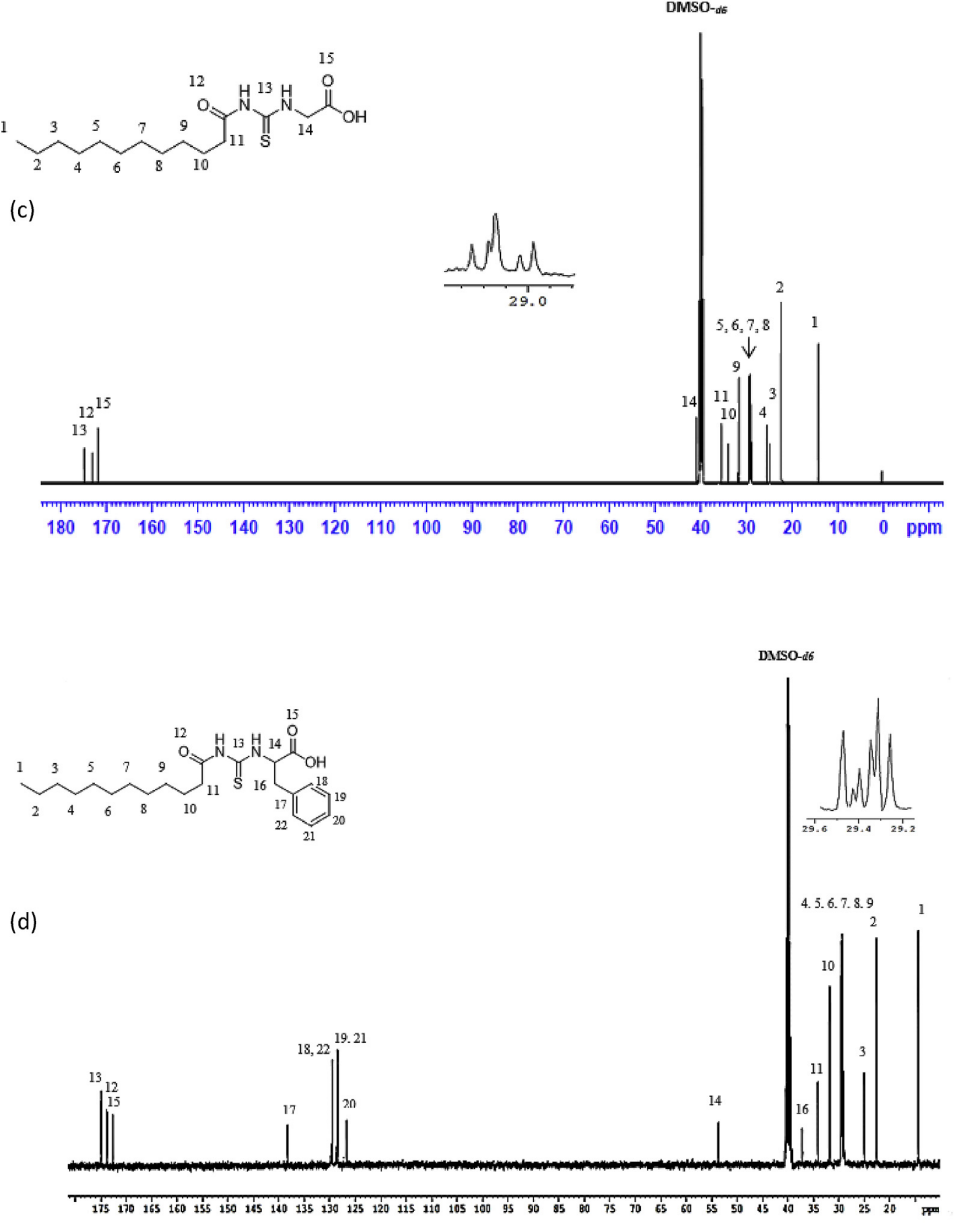
^13^C NMR spectra of (a) R1, (b) R2, (c) R3, (d) R4.

**Fig. 6:**
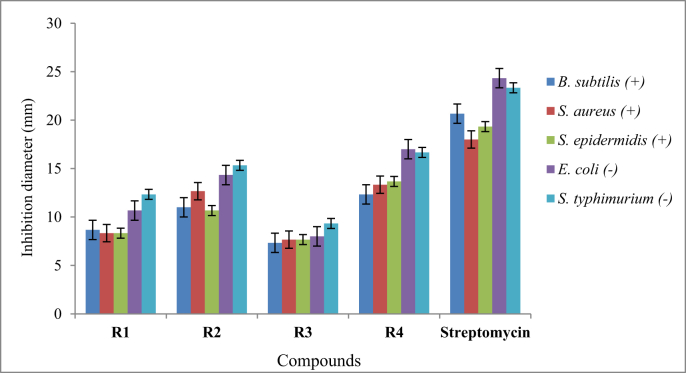
Inhibition diameter of ligands at 1 mg/mL.

Reaction of lauroyl chloride, ammonium thiocyanate and amino acids has led to formation of four new thioureido derivatives namely (3-dodecanoyl-thioureido)propionic acid (R1), 2-(3-dodecanoyl-thioureido)-3-methyl butyric acid (R2), (3-dodecanoyl-thioureido)acetic acid (R3) and 2-(3-dodecanoyl-thioureido)-3-phenyl propionic acid (R4) (Fig. 1). This reaction involves nucleophilic substitutions (SN2), in order to produce lauroyl isothiocyanate as intermediate, before being charged with amino acids [1]. All four compounds were fully characterized by common spectroscopic techniques such as Fourier Transform Infrared (FT-IR), ^1^H and ^13^C Nuclear Magnetic Resonance (NMR) and UV-Visible (Fig. 2, Fig. 3, Fig. 4, Fig. 5). Elemental analysis and spectroscopic data are listed in Table 1, Table 2, Table 3, Table 4, Table 5, respectively. Inhibition diameter of the ligands are illustrated in Fig. 6.

**Table 1:** Elemental analysis (%) of elements present in the compound.

Compound	C	H	N	S
R1	58.10	9.02	8.28	9.54
	(58.15)	(9.15)	(8.48)	(9.70)
R2	60.25	9.47	7.75	8.77
	(60.30)	(9.56)	(7.81)	(8.94)
R3	56.51	8.84	8.70	9.98
	(56.93)	(8.92)	(8.85)	(10.13)
R4	64.79	8.34	6.78	7.80
	(64.99)	(8.43)	(6.89)	(7.89)

*In bracket: Theoretical Calculated Values.

**Table 2:** Summary of the absorption data for R1-R4.

Compound/Stretching	*ν* (N–H) str (cm^−1^)	*ν* (OH) str (cm^−1^)	*ν* (C=O) _COOH_ str (cm^−1^)	*ν* (C=O) NH_2_ str (cm^−1^)	*ν* (C–N) str (cm^−1^)	*ν* (C=S) str (cm^−1^)
R1	NH overlap with OH at 3291 (m)		1695 (s)	1634 (s)	1233 (m)	720 (m)
R2	NH overlap with OH at 3332 (m)		1712 (s)	1650 (s)	1236 (m)	723 (w)
R3	NH overlap with OH 3317 (m)		1701 (s)	1645 (s)	1249 (s)	719 (m)
R4	NH overlap with OH at 3314 (m)		1710 (s)	1692 (s)	1248 (s)	698 (m)

**Table 3:** UV-Vis data for R1-R4.

	Wavelength, (nm)	Absorbance	Assignment
R1	271.00	0.250 (*ε* = 1650 M^−1^cm^−1^)	n-π* & π-π* (C=O) n-π*(C=S)
R2	272.00	0.577 (*ε* = 5770 M^−1^cm^−1^)	n-π* & π-π* (C=O) n-π*(C=S)
R3	269.00	0.194 (*ε* = 330 M^−1^cm^−1^)	n-π* & π-π* (C=O) n-π*(C=S)
R4	275.500	0.416 (*ε* = 4160 M^−1^cm^−1^)	n-π* & π-π* (C=O) n-π*(C=S) π-π* (p-band aryl)

**Table 4:** ^1^H NMR data of R1, R2, R3 and R4.

Compound	Moieties	Chemical Shift, *δ*_H_ (ppm)
R1	(3H, t, *J* = 7.35 Hz, CH_3_)	0.85
	(4H, m, *J* = 6.65 Hz, 2 × CH_2_)	1.43–1.48
	(4H, m, *J* = 5.13 Hz, 2 × CH_2_)	2.00–2.03
	(4H, m, *J* = 7.7 Hz, 2 × CH_2_)	2.17–2.19
	(2H, m, *J* = 7.23 Hz, CH_2_)	2.33–2.36
	(4H, m, *J* = 8.57 Hz, 2 × CH_2_)	2.57–2.59
	(2H, t, *J* = 6.30 Hz, CH_2_)	2.98
	(2H, t, *J* = 5.95 Hz, CH_2_)	3.20
	(2H, t, *J* = 6.3 Hz, CH_2_)	3.82
	(1H, t, *J* = 5.6 Hz, NH)	7.84
	(1H, s, NH)	10.81
	(1H, s, OH)	11.12
R2	(3H, t, *J* = 7.0 Hz, CH_3_)	0.89
	(6H, d, *J* = 7.0 Hz, 2 × CH_3_)	0.97
	(2H, m, *J* = 7.0 Hz, CH_3_)	1.09–1.13
	(6H, m, *J* = 5.8 Hz, 3 × CH_2_)	1.20–1.25
	(2H, m, *J* = 7.9 Hz, CH_2_)	1.64–1.69
	(8H, m, *J* = 5.2 Hz, 4 × CH_2_)	2.34–2.40
	(1H, m, *J* = 4.4 Hz, CH)	2.46–2.48
	(2H, t, *J* = 3.4 Hz, CH_2_)	4.60–4.80
	(1H, d, *J* = 4.2 Hz, CH)	4.99
	(1H, d, *J* = 8.4 Hz, NH)	6.13
	(1H, s, NH)	9.16
	(1H, s, OH)	11.03
R3	(3H, t, *J* = 7.00 Hz, CH_3_)	0.85
	(4H, m, *J* = 7.00 Hz, 2 × CH_2_)	1.49–1.51
	(6H, m, *J* = 7.35 Hz, 3 × CH_2_)	2.09–2.11
	(6H, m, *J* = 7.35 Hz, 3 × CH_2_)	2.17–2.19
	(2H, m, *J* = 5.13 Hz, CH_2_)	2.31–2.33
	(2H, t, *J* = 5.6 Hz, CH_2_)	3.71
	(2H, s, CH_2_)	4.26
	(1H, t, *J* = 5.95 Hz, NH)	8.09
	(1H, s, NH)	9.33
	(1H, s, OH)	9.65
R4	(7H, m, *J* = 4.2 Hz, CH_3_ + 2 × CH_2_)	0.84–0.87
	(2H, m, *J* = 7.0 Hz, CH_2_)	1.07–1.11
	(2H, m, *J* = 6.3 Hz, CH_2_)	1.35–1.39
	(2H, m, *J* = 7.0 Hz, CH_2_)	1.47–1.51
	(2H, m, *J* = 6.0 Hz, CH_2_)	2.00–2.03
	(2H, m, *J* = 6.6 Hz, CH_2_)	2.32–2.36
	(2H, m, *J* = 7.9 Hz, CH_2_)	2.81–2.84
	(2H, m, *J* = 6.3 Hz, CH_2_)	3.03–3.06
	(2H, t, *J* = 7.0 Hz, CH_2_)	3.10
	(2H, d, *J* = 3.5 Hz, CH_2_)	4.42
	(1H, t, *J* = 6.3 Hz, CH)	5.05
	(5H, m, *J* = 7.7 Hz, Ar-H)	7.14–7.28
	(1H, d, *J* = 7.7 Hz, NH)	8.09
	(1H, s, NH)	10.97
	(1H, s, OH)	11.28

**Table 5:** ^13^C NMR data of R1, R2, R3 and R4.

Compound	Moieties	Chemical shift *δ*_C_, (ppm)
R1	(CH_3_)	14.41
	(CH_2_)	22.56
	(CH_2_)	25.71
	(6 × CH_2_)	29.08–29.49
	(CH_2_)	31.76
	(CH_2_–C=O)	34.39
	(CH_2_–COOH)	35.16
	(CH_2_–NH)	35.74
	(C=O–OH)	172.72
	(C=O–NH)	173.37
	(C=S)	174.97
R2	(2 × CH_3_)	14.17
	(CH_3_)	17.70
	(CH_2_)	19.02
	(CH_2_)	24.70
	(CH_2_)	25.77
	(5 × CH_2_)	29.23–29.59
	(CH_2_)	31.72
	(CH)	34.48
	(CH_2_–CO)	36.64
	(CH–NH)	57.21
	(C=O–OH)	174.19
	(C=O–NH)	176.44
	(C=S)	179.94
R3	(CH_3_)	14.42
	(CH_2_)	22.56
	(CH_2_)	24.95
	(CH_2_)	25.64
	(4 × CH_2_)	29.06–29.48
	(CH_2_)	31.76
	(CH_2_)	34.12
	(CH_2_–C=O)	35.52
	(CH_2_–COOH)	40.96
	(C=O–OH)	171.90
	(C=O–NH)	173.08
	(C=S)	174.98
R4	(CH_3_)	14.37
	(CH_2_)	22.60
	(CH_2_)	25.60
	(6 × CH_2_)	29.23–29.52
	(CH_2_)	31.81
	(CH_2_–CO)	34.12
	(CH_2_-Ar)	37.22
	(CH–NH)	53.69
	(C_6_H_5_)	126.69–138.25
	(C=O–OH)	172.58
	(C=O–NH)	173.70
	(C=S)	174.89

## 2 Experimental design, materials, and methods

Lauroyl chloride (3.272 g, 0.015 mol) and ammonium thiocyanate (1.142 g, 0.015 mol) were dissolved in acetone (25 mL) and stirred for an hour to give white precipitate. Then, β-alanine (1.336 g, 0.015 mol) was added dropwise and the mixture was heated at reflux until reaction was completed being filtered into ice. This was monitored by thin layer chromatography. The resulting solid was collected by filtration, washed with acetone and dried under vacuum to give 3-(3-dodecanoyl-thioureido)-propionic acid (R1). The synthesis of 2-(3-dodecanoyl-thioureido)-3-methyl-butyric acid (R2), (3-dodecanoyl-thioureido)-acetic acid (R3) and 2-(3-dodecanoyl-thioureido)-3-phenyl-propionic acid (R4) were prepared in similar manner as described for R1, employing dl-valine, glycine and l-phenylalanine as amino acid. The ligands were prepared following literature method described in the literature [2]. The references are used as guidance in characterizing significant peaks from FTIR [3] and ^1^H and ^13^C NMR spectra [4,5].

The antibacterial activity of compounds R1-R4 were screened against test strains of Gram-positive (*Bacillus subtilis* ATCC 11774, *Staphylococcus epidermidis* ATCC 13518 and *Staphylococcus aureus* ATCC 25923) and Gram-negative (*Escherichia coli* ATCC 11775 and *Salmonella typhimurium* ATCC 14128) strains using common well diffusion method. Mueller-Hinton media were seeded with bacterial inoculum using cotton swab. Wells of 6.0 mm diameter were bored into the media using sterile cork borer and 90 μL of the diluted compounds at a dose range of 10–0.01 mg/mL were added in each well. Streptomycin (Abtek Biologicals Ltd) was used as the positive control while methanol served as negative control. All plates were incubated overnight at 37 °C. The antibacterial activity was evaluated by measuring the zone of inhibition (mm) and minimum inhibitory concentrations (MIC). Ligand that have high specific surface area structure gave higher antibacterial activity [6].
